# Analysis of morbidity and mortality in patients with primary bone tumors who underwent sacrectomy: A systematic review

**DOI:** 10.1016/j.jbo.2022.100445

**Published:** 2022-07-16

**Authors:** Mayara Branco e Silva, Mayara Branco e Silva, Samilly Conceição Maia Martins, Karen Voltan Garofo, Carlos Eduardo Hideo Hanasilo, Mauricio Etchebehere

**Affiliations:** Department of Orthopedics, Rheumatology and Traumatology, Faculty of Medical Sciences, The State University of Campinas (FCM - UNICAMP), Tessalia Vieira de Carvalho Avenue, 126, Campinas 13083-887, Brazil

**Keywords:** Sacrectomy, Morbidity, Mortality, Bone tumors, Chordoma, Sacrum

## Abstract

**Background:**

Sacrectomy is indicated for the resection of life-threatening tumors in the sacrum area. Several studies have been conducted to investigate important aspects of sacrectomy to help reduce the morbidity and mortality of patients who underwent the procedure. This aim of this systematic review was to highlight the prognoses of patients who underwent sacrectomy for the resection of primary bone tumors by analyzing information related to the intraoperative and perioperative periods of the procedure.

**Methodology:**

Several databases were searched for relevant articles using the keywords “sacrectomy” and “survival” associated with the Boolean operators “or” and “and” ([SACRECTOMY OR SACRECTOM*] AND SURVIVAL).

**Results:**

A total of 13 articles were selected for data collection. The studies reported in the articles included a total of 384 patients, 140 of whom underwent partial sacrectomy, whereas 244 underwent total sacral resections. The results of the analysis indicated that the average volume of blood lost during a resection performed using the combined anterior and posterior approaches (average duration, 8.35 h) was 4571.94 mL. Regarding poor outcomes and adverse events in the included studies, 10 patients died in the early postoperative period, whereas four patients had hemorrhagic shock. The most prevalent complications reported were surgical wound infection and sphincter dysfunction.

**Conclusion:**

The optimal surgical approach for sacrectomy depends on the location of the tumor. The anterior approach, preferably with laparoscopy, is currently widely used to reduce the amount of blood lost during the procedure. Although the most prevalent complications of sacrectomy have a high incidence rate, the procedure has a low mortality rate.

## Highlights

• Sacrectomy is indicated for the treatment of benign or malignant primary bone tumors.
• The choice of surgical technique depends on the size, location of the tumor.
• Rate of mortality is low, while massive bleeding was the main cause of death.
• Most prevalent complications are surgical wound infection and sphincter dysfunction.

## 1 Introduction

Sacrectomy is mainly indicated when a life-threatening tumor is detected in the sacrum area [1]. Primary tumors of the sacrum are rare and include benign neoplasms, such as osteochondroma, giant cell tumors, and osteoid osteoma, as well as malignant neoplasms, such as chordoma, chondrosarcoma, osteosarcoma, Ewing's sarcoma, and myeloma [2], [3]. The best disease-free survival of patients who undergo sacrectomy is achieved through en bloc resection of the lesion, which usually involves partial or total resection of the sacrum [4]. Tumors in the sacrum area usually induce mild and transient symptoms because of their slow growth. The occurrence of pain and neurological disorders, which are more severe symptoms, are usually caused by disc protrusion [1], [5]. Owing to their slow progression, sacral lesions are usually already large at the time of diagnosis and are sometimes inoperable, making surgical treatment challenging and causing hesitation in making clinical decisions [1], [6].

Radical and extensive resection of sacral lesions often requires the sacrifice of important structures in the pelvic area, such as the rectum, iliac vessels, and lumbosacral plexus nerves [7]. Therefore, an understanding of the anatomy of this complex region is necessary to minimize the occurrence of sequelae from the procedure [1], [8], [9]. In addition, the surgical margin should not be compromised to preserve structures, since disease-free survival requires tumor-free margins [6], [10].

The choice of surgical technique for sacrectomy depends on the size, location, and histological type of the tumor. Sacrectomy can be performed using an anterior-only, anterior and posterior, or a posterior-only approach [6], [11], [12]. Margin involvement and intralesional curettage can increase the rate of recurrence and reduce the survival rate of patients. Therefore, wide lesion resection is the most effective technique for controlling and reducing the risk of local recurrence of primary sacral tumors and prolonging a patient's life [6], [13], [14], [15], [16], [17].

Several studies have been conducted to clarify various aspects of sacrectomy, with the aim of reducing the morbidity and mortality rates of the procedure. Since a multidisciplinary team, including oncologists, radiologists, pathologists, cancer surgeons, and spine surgeons, is necessary for a complete sacrectomy procedure [4], [18], the morbidity and mortality outcomes of the procedure depend on which sacral roots are sacrificed to achieve a wide margin and on the level at which the procedure is performed [1]. Infection, massive hemorrhage, surgical wound infection, flap necrosis, and sphincter and neurological dysfunction are the main complications associated with sacrectomy [1], [19], [20], [8], [16]. These complications are related to the increased duration of surgery, the surgical approach used, the amount of blood lost during the procedure, and the sacral roots preserved [1], [5].

The aim of this systematic review was to collate and analyze information related to the intraoperative and perioperative periods of sacrectomy performed for the resection of primary bone tumors to further clarify the prognoses of patients who underwent the procedure and to facilitate better management of patients during the above mentioned. Given the rarity of sacral primary bone tumors, the primary goal was to better inform all the medical personnel involved in related procedures.

## 2 Methods

This systematic review was performed according to the Preferred Reporting Items for Systematic Reviews and meta-Analyses recommendations [21]. The Patient, Intervention, Comparison and Outcomes (PICO) methodology was used to define the clinical question [22]. In PICO, “P” corresponds to the population included in the studies (patients with primary bone tumors of the sacral region), “I” defines the intervention to be investigated (total or partial sacrectomy), “C” denotes the comparison between the studies and their results regarding the complications and outcomes associated with the procedure, and “O” defines the investigated outcomes (the conclusion of the studies regarding the therapies): What is the estimated surgical time needed for the approach? Control of intraoperative bleeding? Main complications? Re-approach needs? Predicted neurological deficits? Is it related to the histological tumor type? Does the lesion resection level influence it? Does the tumor size at its largest diameter have an influence?

For this review, the BVS-Bireme, PUBMED, PUBMEDPMC, SCOPUS, WEB OF SCIENCE, EMBASE, COCHRANE LIBRARY, PROQUEST, and EBSCOHost databases were searched for relevant articles using the keywords “sacrectomy” and “survival” associated with the Boolean operators “or” and “and” ([SACRECTOMY OR SACRECTOM*] AND SURVIVAL). No limit regarding the year of publication was set, and the final search date was updated to March 2020.

**Fig. 1:**
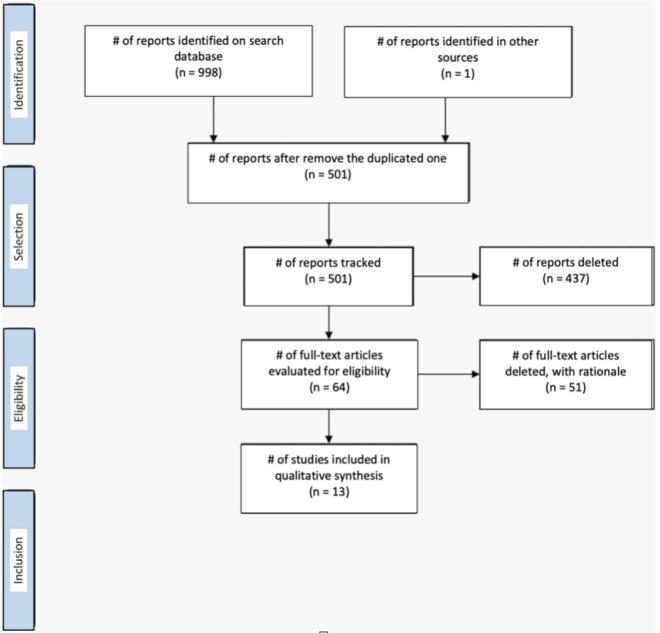
Flowchart of the different phases of the systematic review.

A total of 998 articles were retrieved through the database search. Of these, 458 duplicates in Endnote and another 40 articles identified as duplicates in the Rayyan system were excluded, leaving a total of 501 articles for analysis. Two reviewers initially evaluated the titles and abstracts of the articles identified from the electronic search. The titles and abstracts were reviewed according to the inclusion and exclusion criteria. Conflicts were discussed among reviewers and resolved by consensus of the analysts. After the titles and abstracts were reviewed, systematic reviews, case reports, descriptions of surgical techniques, articles on sacrectomy performed using secondary implants, and articles missing the necessary intraoperative and perioperative data for analysis were excluded. A total of 64 articles remained after exclusion. Of these, 12 articles met the inclusion criteria for the systematic review. Another article was added after the full texts of the articles were read and their bibliographies analyzed (Fig. 1).

## 3 Results

### 3.1 A total of 13 articles were analyzed for data collection. The details of the articles are shown in

Table 1. Complete analysis of the articles showed that most of the studies reported were focused on the treatment of patients with chordoma due to its high local recurrence rate. As shown in Table 2, which contains the histological profiles of the primary bone tumors treated in the studies using sacrectomy, four studies [23], [27], [28], [29], [30] involved the analysis of patients with sacral chordoma. Of the 384 patients included in the 13 analyzed studies, 268 had chordomas, 30 had giant cell tumors, 23 had chondrosarcoma, 19 had Ewing's sarcoma, 15 had osteosarcoma, and 29 had other pathologies.

**Table 1:** Included studies on morbidity and mortality of sacrectomy.

Sources	Article Title	PUBLICATION YEAR
ASAVAMONGKOLKUL ET AL. [23]	WIDE RESECTION OF SACRAL CHORDOMA VIA POSTERIOR APPROACH	2012
ARKADER ET AL. [24]	HIGH LONG-TERM LOCAL CONTROL WITH SACRECTOMY FOR PRIMARY HIGH-GRADE BONE SARCOMA IN CHILDREN	2012
GARCIA ET AL. [25]	*TUMORES PRIMARIOS DE SACRO: ANÁLISIS DE RESULTADOS Y COMPLICACIONES*	2018
DANG ET AL. [26]	PROGNOSIS AND RISK FACTOR INFLUENCING RECURRENCE IN SURGERY - TREATED PATIENTS WITH PRIMARY SACRAL TUMORS	2017
DUBORY ET AL. [27]	“EN BLOC” RESECTION OF SACRAL CHORDOMAS BY COMBINED ANTERIOR AND POSTERIOR SURGICAL APPROACH: A MONOCENTRIC RETROSPECTIVE REVIEW ABOUT 29 CASES	2014
HULEN ET AL. [28]	ONCOLOGY AND FUNCTIONAL OUTCOME FOLLOWING SACRECTOMY FOR SACRAL CHORDOMA	2006
DUBORY ET AL. [29]	INTEREST OF LAPAROSCOPY FOR EN BLOC “RESECTION OF PRIMARY MALIGNANTE SACRAL TUMORS BY COMBINED APPROACH: COMPARATIVE STUDY WITH OPEN MEDIAN LAPAROTOMY	2015
SOLINI ET AL. [30]	EMISACRECTOMY, EXPERIENCE IN 11 CASES	2009
VERLAAN ET AL. [31]	COMPLICATION, SECONDARY INTERVENTIONS AND LONG-TERM MORBIDITY AFTER EN BLOC SACRECTOMY	2015
ZANG ET AL. [32]	IS TOTAL EN BLOC SACRECTOMY USING A POSTERIOR-ONLY APPROACH FEASIBLE AND SAFE FOR PATIENTS WITH MALIGNANT SACRAL TUMORS?	2015
ZHANG ET AL. [6]	PRELIMINARY INVESTIGATION OF BILATERAL INTERNAL ILIAC ARTERY LIGATION AND ANTERIOR TUMOR SEPARATION THROUGH LAPAROSCOPY BEFORE POSTERIOR RESECTION OF GIANT SACRAL TUMOR	2020
ZILELI ET AL. [8]	SURGICAL TREATMENT OF PRIMARY SACRAL TUMORS: COMPLICATIONS ASSOCIATED WITH SACRETOMY	2003
LI ET AL. [33]	SURGICAL CLASSIFICATION OF DIFFERENT TYPES OF EN BLOC RESECTION FOR PRIMARY MALIGANT SACRAL TUMORS	2011

**Table 2:** Histopathological profile of primary bone tumors.

SOURCES	TOTAL	CHORDOMA	GCT	CHONDROSARCOMA	EWING’S SARCOMA	OSTEOSARCOMA	OTHERS
ASAVAMONGKOLKUL ET AL. [23]	21	21	–	–	–	–	–
ARKADER ET AL. [24]	8	–	–	–	6	2	–
GARCIA ET AL. [25]	22	13	3	1	1	1	3
DANG ET AL. [26]	56	37	13	–	1	3	2
DUBORY ET AL. [27]	29	29	–	–	–	–	–
HULEN ET AL. [28]	16	16	–	–	–	–	–
DUBORY ET AL. [29]	33	31	–	–	1	–	1
SOLINI ET AL. [30]	11	9	–	–	–	–	2
VERLAAN ET AL. [31]	16	7	–	5	–	–	4
ZANG ET AL. [32]	10	4	1	1	2	1	1
ZHANG ET AL. [6]	34	18	7	1	–	1	7
ZILELI ET AL. [8]	11	7	–	3	–	1	–
LI ET AL. [33]	117	76	6	12	8	6	9
**TOTAL**	**384**	**268**	**30**	**23**	**19**	**15**	**29**

Due to the low incidence of sacral tumors, the studies involved long-term hospital analyses. The shortest period evaluated was 2 years (2011–2013), which was evaluated in a Chinese study on the safety of utilizing only the posterior approach for resection of malignant tumors [32]. The longest period studied was 29 years (1985–2014), which was evaluated in a study of intraoperative assessment before and after the introduction of laparoscopy in the anterior approach of sacrectomy [29].Analysis of the articles indicated that of the 384 procedures performed, 140 were partial sacrectomies (resection at S3 or below) and 244 were total sacrectomies (resection above S2). Total sacral resection is an important parameter to be analyzed since loss of pelvic stability above level S2 requires reconstruction, which can range from installing iliac screws to spine-pelvic fixation with pedicle screws in the lumbar vertebrae. Such a need influences the amount of intraoperative blood loss and the surgical time required for tumor resection.

Surgical wound infection and the need for a follow-up procedure for debridement were the most significant complications reported in the studies. Of the 384 procedures performed, approximately 28 % of the cases required at least one surgical procedure for debridement. Another relevant complication was sphincter control soon after the procedure (313 [81 %] patients with urinary disorders and 273 [71 %] fecal incontinence), shown in (Table 3). Some more severe complications were reported in the studies as well. Verlaan et al. [31] reported a case of meningitis and a case of sacral fracture, whereas Dang et al. [26], Zhang et al. [6], and Zileli et al. [8] reported cases of cerebrospinal fluid fistula.

**Table 3:** Main post-operative complications.

SOURCES	WOUND INFECTION	WOUND DEHISCENCE	DEBRIDEMENT	URINARY DISORDER	FECAL INCONTINENCE
ASAVAMONGKOLKUL ET AL. [23]	0	0	3*	21	12
ARKADER ET AL. [24]	4	3	4	8	8
GARCIA ET AL. [25]	8	0	8	22	15
DANG ET AL. [26]	2	9	5	23	23
DUBORY ET AL. [27]	18	7	18	29	29
HULEN ET AL. [28]	8	12	12	15	15
DUBORY ET AL. [29]	19	8	NI	12	16
SOLINI ET AL. [30]	1	3	1	11	11
VERLAAN ET AL. [31]	12	10	13	14	11
ZANG ET AL. [32]	3	1	3	10	10
ZHANG ET AL. [6]	7	3	10	22	11
ZILELI ET AL. [8]	5	0	NI	11	11
LI ET AL. [33]	31	0	31	115	101
TOTAL	**118**	**56**	**108**	**313**	**273**

Some authors evaluated the ability of patients to walk after sacrectomy. The data from the seven studies in which this parameter was evaluated shows that about two thirds of patients were able to walk without assistance, whereas only 11 patients were unable to walk during the postoperative period and needed a wheelchair for locomotion.

All the articles included information on patients who died in the early postoperative period. A total of 10 deaths were recorded, mostly due to hemorrhagic shock. Sepsis due to rectal perforation and pulmonary thromboembolism were also common causes of death.

Since blood loss was the most common cause of death, it was essential to analyze the volume of blood loss reported in the studies. The volume of intraoperative blood loss was quite significant, especially in cases where a combined surgical approach was needed (Table 4). Asavamongkolkul et al. [23] and Zang et al. [32] evaluated the safety of using only the posterior approach when performing resection of malignant bone tumors and reported a mean blood loss of 905.5 mL and 2595 mL, respectively. However, most of the articles do not specifically indicate the volume of blood lost at each step of the procedure. Despite this, it was possible to estimate that the mean volume of blood lost during sacrectomy using the anterior and posterior approach was 4571.94 mL. Zhang et al. [6] evaluated the use of laparoscopy as an anterior approach to isolate the sacral tumor from the structures anterior to it, and ligature of the bilateral internal iliac veins following with the posterior approach to complete the tumor resection. They reported an average total blood loss of 1757.64 mL, a significant reduction in the mean blood volume lost during the procedure. Dubory et al. [29] compared the use of laparotomy and laparoscopy for the anterior approach and also reported that the volume of blood lost using laparoscopy for the anterior approach was less than that lost using laparotomy. Another variable of major interest was the duration of surgery, as it is related to increase in the rate of infection and the need for multiple teams to complete the procedure. Analysis of the included studies showed that the duration of sacrectomy performed using the posterior approach was longer than that performed using the anterior approach. The results also showed that the average duration of the combined anterior and posterior approach was 8.35 h (Table 5). However, the duration of surgery was not as relevant to the outcome as the volume of blood loss during sacrectomy using the laparoscopic approach, As evidenced by the results of the studies by ASAVAMONGKOLKUL et al. [23] and ZANG et al [32].

**Table 4:** Bleeding volume evaluated according to the surgical approaches used (mL).

SOURCES	ANTERIOR	POSTERIOR	ANTERIOR + POSTERIOR
ASAVAMONGKOLKUL ET AL. [23]	0	905,5	0
ARKADER ET AL. [24]	NI	NI	7000
GARCIA ET AL. [25]	NI	NI	2100
DANG ET AL. [26]	1984,62	2162,50	2633,33
DUBORY ET AL. [27]	894,6	3285	4196.7
HULEN ET AL. [28]	NI	NI	5000
DUBORY ET AL. [29]	2208,3	NI	5385,7
SOLINI ET AL. [30]	NI	NI	1500
VERLAAN ET AL. [31]	NI	NI	12,000
ZANG ET AL. [32]	NI	2595	0
ZHANG ET AL. [6]	NI	NI	175,64*
ZILELI ET AL. [8]	NI	NI	4518
LI ET AL. [33]	NI	2300	4200
**MEAN**	1551,45	1593,257	4571,943

*anterior approach by laparoscopy; NI(not informed).

**Table 5:** Surgical time according to the surgical approach used (H).

SOURCES	ANTERIOR	POSTERIOR	ANTERIOR + POSTERIOR
ASAVAMONGKOLKUL ET AL. [23]	0	7.25	0
ARKADER ET AL. [24]	NI	NI	19
GARCIA ET AL. [25]	NI	NI	3.81
DANG ET AL. [26]	2.37	2.99	5.21
DUBORY ET AL. [27]	3.71	3.75	7.5
HULEN ET AL. [28]	NI	NI	16
DUBORY ET AL. [29]	NI	NI	7.62
SOLINI ET AL. [30]	NI	NI	9
VERLAAN ET AL. [31]	NI	NI	12.7
ZANG ET AL. [32]	NI	4.7	0
ZHANG ET AL. [6]	1.27	3.33	4.6
ZILELI ET AL. [8]	NI	NI	13.4
LI ET AL. [33]	NI	3.1	9.8
**MEAN**	**1.83**	**4.18**	**8.35**

NI:(not informed).

A total of 211 wide surgical margins were reported in the studies, which is a relevant number even without the information from two [8], [26] of the 13 articles analyzed. This data is also relevant considering the difficulty of achieving free margins since sacral tumors are usually close to important structures [1], [7], [34].

## 4 Discussion

In this systematic review, we analyzed information related to the intraoperative and perioperative periods of the sacrectomy to clarify the prognoses patients who underwent the procedure. Sacrectomy is the procedure indicated for treating primary sacral bone tumors. The approach for the procedure depends on the histological type, size, and location of the tumor, it could be anterior-only, anterior and posterior, or a posterior-only approach [6], [11], [12]. However, the combined approach is still widely used, especially when sacrectomy involves the proximal portions of the sacrum. Nonetheless, the combined approach had an average of 4.5 L of blood loss and 8 h surgery time, and an especially high morbidity rate regarding sexual and sphincter dysfunctions.

The authors of the analyzed studies favor laparoscopy for the anterior approach, which significant data has shown to be associated with less blood loss than the laparotomy approach without significant changes in the duration of the entire procedure [6], [29].

The treatment of sacral bone tumors is quite challenging as a wide surgical margin is required for the treatment of both benign and malignant tumors to obtain satisfactory results. However, neurovascular structures and functions associated with this region, such as urological, anal-rectal, and sexual, can be sacrificed during sacrectomy to obtain this margin [1], [19], [20]. The present study shows that sacrectomy is associated with a high rate of dysfunction, mainly urinary and rectal, especially in the early postoperative days. In addition, the higher the sacral osteotomy level, the higher the number of sacral roots involved, which may increase the chances of sphincter involvement and sexual dysfunction, especially if the roots above S3 are resected close to the tumor [28]. However, sexual dysfunctions were not assessed in the present study owing to the scarcity of relevant data in the analyzed studies regarding this complication.

Although the rate of mortality from sacrectomy is low, massive bleeding was the most prevalent cause of death in the analyzed studies. The rate of complications was high, with surgical wound infection being the most prevalent, sometimes requiring debridement. Sciubba et al. [35] reported that surgical site infection is the most prevalent sacrectomy postoperative complication that can be prevented. Decrease in surgical time, the number of clinical staff treating the surgical wound, and blood loss are factors related to decrease in the rate of infection related to sacrectomy. Similar findings were reported by Li et al. [33] in their study of risk factors for surgical wound infection after sacrectomy.

This study had some limitations due to the rareness of primary sacral bone tumors. It is paramount that researchers keep track and publish studies on the subject to raise awareness in the medical community and improve patient outcomes.

## 5 Conclusion

Sacrectomy is indicated for the treatment of benign or malignant primary bone tumors. The surgical approach for sacrectomy largely depends on the location of the tumor location. However, the anterior approach, preferably through laparoscopy, is currently widely used to reduce the amount of blood lost during the procedure. The most prevalent complications of sacrectomy are surgical wound infection and sphincter dysfunction. Although these complications have a high incidence rate, the procedure has a low mortality rate.

## Declaration of Competing Interest

The authors declare that they have no known competing financial interests or personal relationships that could have appeared to influence the work reported in this paper.
